# Preparation and Evaluation of Transdermal Drug Delivery System of Etoricoxib Using Modified Chitosan

**DOI:** 10.4103/0250-474X.44593

**Published:** 2008

**Authors:** A. Wahid, B. K. Sridhar, S. Shivakumar

**Affiliations:** Department of Pharmaceutics. National College of Pharmacy, Shimoga-577 201, India

**Keywords:** Etoricoxib, chitosan, polymer A, polymer B, chitosan/HPMC blend

## Abstract

In the present investigation chitosan has been chemically modified by treating with two different aldehydes like acetaldehyde and propionaldehyde to form Schiff’s bases. Schiff’s bases of chitosan with acetaldehyde and propionaldehyde were named as polymer A and polymer B, respectively. Fourier Transform Infra Red (FTIR) spectral data have confirmed the reaction carried out on chitosan. Drug free polymeric films of chitosan, chemically modified chitosan and chitosan/hydroxypropylmethylcellulose blend were prepared and evaluated for various physicochemical characters. Further, the films were incorporated with anti-inflammatory drug, etoricoxib using glycerol as plasticizer. The drug loaded films were cross-linked with sodium citrate and studied for permeation characteristics across dialysis membrane and rat skin. All the films were evaluated for bursting strength, swelling index, moisture uptake, thickness uniformity, drug content uniformity, tensile strength, percent elongation at break, percent flatness, water vapour transmission rate and *in vitro* drug permeation study.

The polymeric technologies have been honed and refined over the past several years and currently great interest has been focused on the development of novel drug delivery systems^1^. Treatment of chronic diseases such as asthma, rheumatoid arthritis by transdermal route of drug administration might prove to have several advantages over other routes of drug administration^2^. Chitosan is a natural occurring polymer that exhibits biological properties such as nontoxicity, biocompatibility and low chemical reactivity. Chitosan is insoluble in water but soluble in acidic water. Films produced by casting chitosan solution when applied on the skin cause irritation due to the presence of traces of acid. Hence it is necessary to wash the films repeatedly to remove any traces of acid^1^. Etoricoxib is a nonsteroidal anti-inflammatory drug (NSAID) that exhibits anti-inflammatory, analgesic, and antipyretic activities. Etoricoxib is a potent, orally active, highly selective cyclooxygenase-2 (COX-2) inhibitor within and above the clinical dose range^3^. In this study we report the possible usage of chitosan, chemically modified chitosan and chitosan/HPMC blend for transdermal drug delivery system of etoricoxib.

## MATERIALS AND METHODS

Chitosan was obtained as gift sample form Central Institute of Fisheries Technology, Cochin, India. Acetaldehyde, propionaldehyde and acetic acid were purchased from S. D. Fine Chemicals, Mumbai, India. Gift sample of etoricoxib were obtained from Anglo-French Drugs and Industries Ltd., Bangalore, India. HPMC was obtained from S. D. Fine Chemicals, Mumbai, India. Distilled water was used throughout the study.

### Chemical modification of chitosan:

Chitosan (2% w/v) solution was prepared by dissolving the polymer in 1% acetic acid solution prepared in distilled water. After ensuring complete dissolution of chitosan, 50 ml of the solution was stirred with 2 g of either acetaldehyde (to prepare polymer A) or propionaldehyde (to prepare polymer B). Stirring was continued for 3 h at 60°. Later, the polymer solution was added to acetone to precipitate the chemically modified chitosan^2^.

### Fabrication of blank films:

Solution of polymer A, polymer B, plain chitosan and chitosan/HPMC blend were prepared by dissolving in 1.0% w/v acetic acid solution, respectively. The above solution (15 ml) was poured into a petridish (44.15 cm^2^) precoated with polyureathane and kept in an oven at 40° for complete drying. Films produced were washed with 50% v/v ethanol to remove surface bound traces of acid. The dried films were removed from the petridish and stored in a desiccator until use (Table 1).

**TABLE 1:** COMPOSITION OF VARIOUS BLANK FORMULATIONS

Formulation Code	Plain Chitosan % w/v	Polymer-A % w/v	Polymer-B % w/v	Chitosan/HPMC % w/v
F1	1.5	-	-	-
F2	2	-	-	-
F3	2.5	-	-	-
F4	-	1.5	-	-
F5	-	2	-	-
F6	-	2.5	-	-
F7	-	-	1.5	-
F8	-	-	2	-
F9	-	-	2.5	-
F10	-	-	-	50:50
F11	-	-	-	25:75
F12	-	-	-	75 : 25

### Evaluation of physical and mechanical properties of blank films:

The blank films prepared were evaluated for uniformity of thickness, percent flatness^4^, swelling index^5^, bursting strength, water vapour transmission rate (WVTR)^2^,^6^, percent elongation at break and tensile strength^7^ and moisture uptake^8^.

### Fabrication of drug loaded films:

Solution of polymer A, polymer B, plain chitosan and chitosan/HPMC blend were prepared by dissolving 2 g of polymer in 100 ml of 1.0% w/v acetic acid solution. To the above prepared polymeric solution, 20% w/w, 30% w/w (with respect to dry weight of polymer) of glycerol followed by 20% w/w (with respect to dry weight of polymer) of etoricoxib was added and stirred for 30 min. Drug containing polymeric solution (22 ml) were poured into a petridish (44.15 cm^2^), precoated with polyureathane and kept in an oven at 40° for complete drying. Films produced were washed with 50% ethanol to remove surface bound traces of acid. The dried films were removed from the petridish and stored in a desiccator until use (Table 2).

**TABLE 2:** COMPOSITION OF VARIOUS DRUG LOADED FILMS

Formulation Code	Polymeric Solution % w/v	Polymer/s	Plasticizer * (Glycerol) %	Drug * %
D1	2.0	Plain Chitosan	20.0	20.0
D2	2.0	Plain Chitosan	30.0	20.0
D3	2.0	Polymer A	20.0	20.0
D4	2.0	Polymer A	30.0	20.0
D5	2.0	Polymer B	20.0	20.0
D6	2.0	Polymer B	30.0	20.0
D7	2.0	PlainChitosan + HPMC (75:25)	20.0	20.0
D8	2.0	PlainChitosan + HPMC (75:25)	30.0	20.0

* w/w of dry weight of polymer

### Preparation of cross-linked films:

Cross linking of films was done by dipping in a 10 ml solution of sodium citrate (10% w/v, adjusted to pH 5) for 1h. These films were washed with water to remove excess sodium citrate^9^. The drug loaded cross linked films were assigned code C1 to C8.

### *In vitro* permeation studies using dialysis membrane:

The diffusion cell was fabricated with the help of small funnel. The donor compartment was funnel of diameter 3.5 cm. The transdermal patch of area 1.5 cm^2^ was placed on dialysis membrane with aluminum foil as backing membrane, which was then tied to the diffusion cell. This diffusion cell was immersed in beaker (receptor compartment) containing 50 ml of phosphate buffer of pH 7.4 with methanol 50% v/v as diffusion media. The receptor compartment was stirred by using magnetic stirrer at 100 rpm and the whole assembly was maintained at 37±1°. The amount of drug release was determined by withdrawing 2 ml of sample at specific time intervals upto 12 hrs. The volume withdrawn was replaced with equal volume of fresh and pre-warmed (37°) phosphate buffer media containing methanol. The absorbance of the withdrawn sample was measured after suitable dilution at 235 nm to estimate etoricoxib. The experiment was carried out in triplicate and average value was reported^10^.

### *In vitro* skin permeation studies:

The *in vitro* skin permeation studies were carried out using dorsal section of full thickness skin from albino rats (weighing between 200–250 g) whose hair had been removed on the previous day using electric clipper. The transdermal patches were firmly pressed on the centre of the rat skin. Once adhesion to the skin surface had been confirmed, the skin was quickly mounted on the diffusion tube which acted as the donor compartment. 50 milliliter of phosphate buffer of pH 7.4 with methanol 50% v/v as diffusion media was taken in a beaker, which acted as the receptor compartment to maintain the sink condition. The donor compartment was kept in contact with the receptor compartment and the receptor compartment was stirred magnetically during the study. After every 1 h, sample (2 ml) was withdrawn and replaced with 2 ml of fresh phosphate buffer pH 7.4 with methanol 50% v/v. The samples were then analyzed using UV spectrometer at 235 nm to estimate etoricoxib.

## RESULTS AND DISCUSSION

**Fig. 1:**
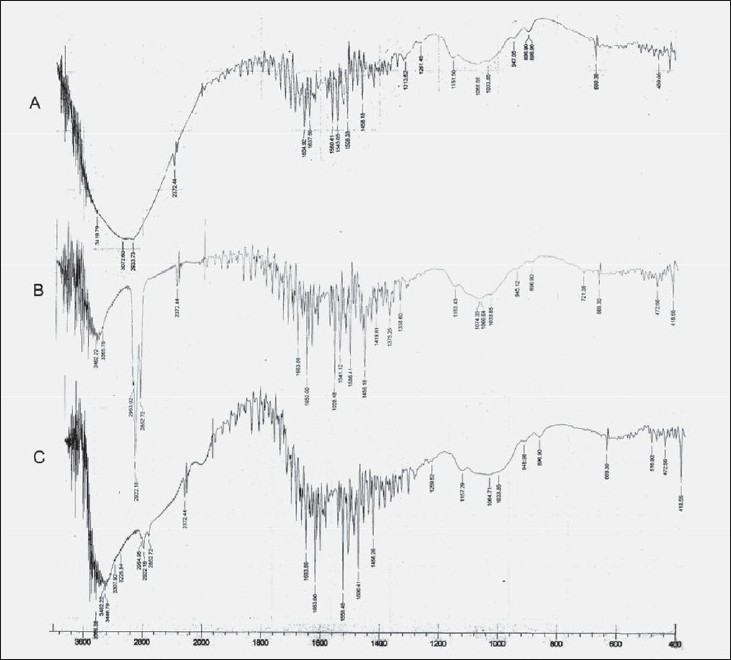
FTIR Spectrum of blank polymeric films: A: chitosan, B: chitosan modified with acetaldehyde and C: chitosan modified with propionaldehyde

**Fig. 2:**
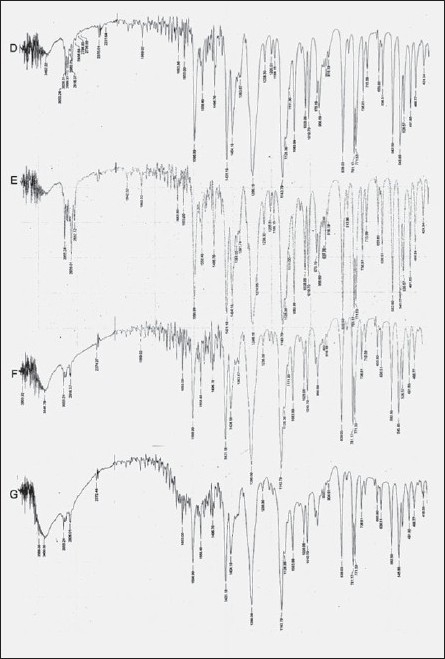
FTIR Spectrum of drug loaded polymeric films. D: etoricoxib, E: chitosan modified with acetaldehyde with drug, F: chitosan modified with propionaldehyde with drug and G: chitosan with drug

The free amino group of chitosan was reacted with aldehyde in presence of acid to form Schiff’s base. Aldehydes were selected based on their film forming capacity with the polymer. The percent aldehyde conversion and carbon chain length of the aldehyde, affected the film characteristics. The FTIR spectrum of plain chitosan, polymer A and polymer B was taken to confirm the modification of chitosan and stability of drug. Chitosan showed peak at 1637 cm^-1^ corresponding to amino groups. In contrast, after formation of imino group (-C=N-) a new peak appeared at 1541 cm^-1^ for polymer A and at 1558 cm^-1^ for polymer B (fig. 1). FTIR of pure etoricoxib and drug loaded membranes were also obtained to find out if there was any chemical interaction between drug and the polymer. Etoricoxib showed characteristic peak at 1143 cm^-1^ corresponding to sulphone groups (-S=O) and did not alter even after loading into the membrane, this confirms stability (no interaction) of the drug (fig. 2). FTIR of HPMC and chitosan/HPMC blend were obtained to find out if there was any interaction between HPMC and chitosan. FTIR of drug loaded chitosan/HPMC blend film was also obtained to evaluate the chemical interaction between drug and chitosan/HPMC blend. Etoricoxib characteristic peak at 1143 cm^-1^ remained unchanged which confirmed the stability of drug.

All the blank films were found to be uniform in thickness. The percent flatness of blank films ranged from 97.40% to 100% flatness. Formulation F2, F5, F8 and F11 showed 100% flatness where as formulation F7 showed percent flatness of 97.40%. Formulation, F2 showed highest bursting strength of 4.3 kg/cm^2^ where as formulation F11 showed low bursting strength of 2.0 kg/cm^2^. Similarly, formulation F6 showed highest water vapour transmission (WVTR) of 10.38×10^-3^ g/cm.day was as formulation F8 showed low moisture uptake of 3.53×10^-3^ g/cm.day. The increase in moisture uptake was observed with increase in relative humidity for all the blank films. All the blank formulation showed increase in weight with time, formulation F3 had highest swelling index of 97.82% when compared to F4 which exhibited low swelling index of 91.50%. Similarly formulation F11 showed highest tensile strength of 5.893 kg/cm^2^ with percent elongation of 50%, formulation F4 show low tensile strength of 2.6122 kg/cm^2^ with percent elongation of 20%. Based on the physical and mechanical properties of the blank films, four best formulations were selected for incorporation of drug. From the above blank films formulation F2, F5, F8 and F12 were selected for incorporation of drug.

All the drug loaded films were found to be quite uniform in thickness. The percent flatness of drug loaded films was ideal (Table 3). All films showed an increase in moisture uptake with an increase in relative humidity. The increase in moisture uptake may be attributed to the hygroscopic nature of polymer-glycerol composite film. All the films showed increase in weight with time. The films with modified polymer showed low swelling index as compared to that of films with plain chitosan (Table 4). Different formulation showed different water vapour transmission rate. (Table 3). The bursting strength has linear correlation with increase in concentration of plasticizer (Table 3). It was noticed that formulation D8 showed highest tensile strength of 4.728 kg/cm^2^ (Chitosan/HPMC blend with 30% glycerol) where as formulation D2 showed 4.258 Kg/cm^2^. Formulation D3 (modified with acetaldehyde with 30% glycerol) showed low tensile strength of 2.690 kg/cm^2^. Formulation D2, D4 and D8 showed highest percent elongation of 80% where as formulation D5 shows low percent elongation of 40%.

**TABLE 3:** AVERAGE THICKNESS, PERCENT FLATNESS, BURSTING STRENGTH AND WATER VAPOUR TRANSMISSION RATE OF FORMULATIONS

Formulation Code	Average Thickness (mm)	Bursting Strength (Kg/cm^2^)	Percent Flatness (%)	Water Vapor Transmission Rate (g/cm.day)
D1	0.166±0.0559	3.8±0.042	99.50	7.9×10^-3^
D2	0.186±0.0352	4.2±0.021	100.00	17.03×10^-3^
D3	0.262±0.0277	2.2±0.015	98.50	25.1×10^-3^
D4	0.196±0.0433	3.8±0.036	99.60	22.0×10^-3^
D5	0.212±0.0248	2.1±0.017	99.00	20.8×10^-3^
D6	0.286±0.0181	2.5±0.009	100.00	28.9×10^-3^
D7	0.198±0.0408	2.2±0.039	98.40	18.1×10^-3^
D8	0.192±0.0164	2.3±0.036	100.00	19.5×10^-3^

**TABLE 4:** PERCENT MOISTURE UPTAKE AT DIFFERENT RH AND SWELLING INDEX FOR DRUG LOADED FILMS

Formulation Code	Percent Moisture Uptake at Different RH			%Swelling Index			
		
	52%	74.9%	98%	5min	10min	30min	60min
D1	8.16	14.0	17.76	73.56	75.97	77.16	78.75
D2	12.85	17.47	18.24	46.94	48.91	49.84	53.78
D3	10.09	18.64	20.53	44.73	46.61	47.71	48.78
D4	12.39	19.87	20.15	33.01	37.44	38.26	40.58
D5	8.16	15.00	17.50	18.18	21.96	25.41	37.50
D6	9.89	16.92	20.31	23.24	27.98	29.71	30.83
D7	12.03	15.60	24.34	62.45	66.54	67.72	70.43
D8	9.85	16.34	24.30	63.32	67.12	68.47	72.54

The selected formulations loaded with etoricoxib were subjected to *invitro* drug release. *Invitro* diffusion studies across dialysis membrane and rat skin was conducted using diffusion cell fabricated with the help of funnel and beaker assembly.

**Fig. 3:**
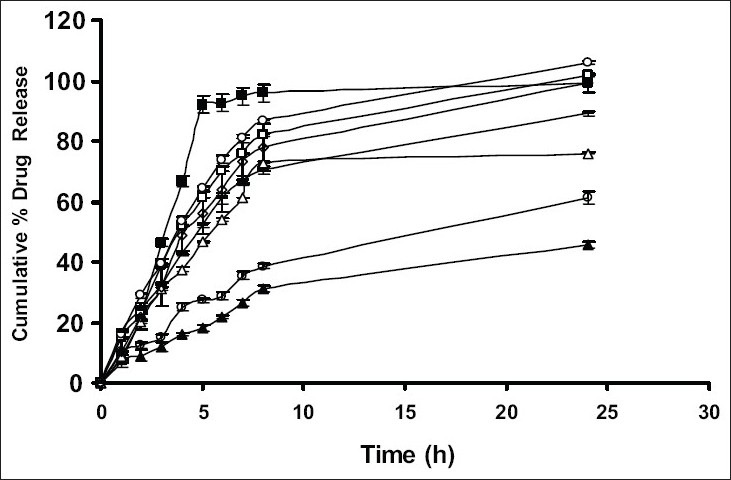
*In vitro* cumulative percent drug release studies of formulation without cross-linking. D1 (-■-), D2 (-□-), D3 (-▲-), D4 (-△-), D5 (-●-), D6 (-○-), D7 (---) and D8 (-◊-)

**Fig. 4:**
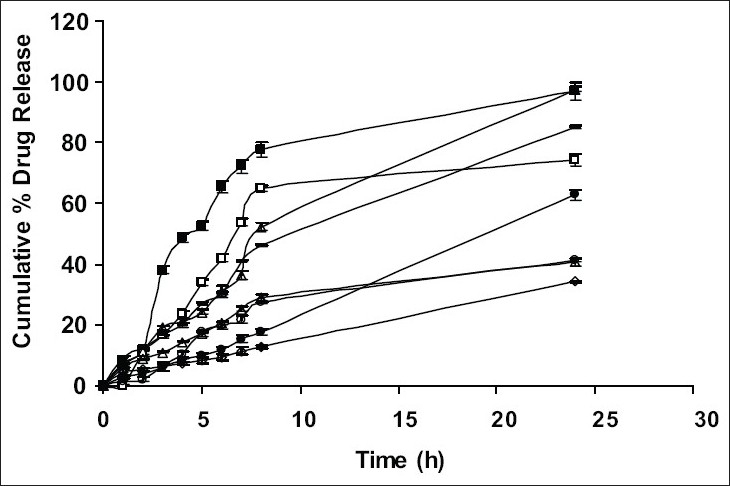
*In vitro* cumulative percent drug release studies of formulation cross-linked with sodium citrate. C1 (-■-), C2 (-□-), C3 (-▲-), C4 (-△-), C5 (-○-), C6 (-●-), C7 (---) and C8 (-◊-)

Drug release from swellable and erodible hydrophilic matrix can be attributed to polymer dissolution (matrix erosion mechanism), drug diffusion through the gel layer or combination of both. When the data was plotted as cumulative percentage of drug permeated versus time (figs. 3 and 4), the data for cumulative percentage drug release were depicted in Table 5. The data were subjected to first order equation and the regression value was found to be in the range of (R^2^ = 0.8825 − 0.9987) which confirm first order release pattern. Further to find out whether diffusion is involved in the drug release, the data was subjected to Higuchi’s equation. The lines obtained were comparatively linear (R^2^ = 0.8486−0.9765) suggesting the diffusion may be mechanism of drug release. To confirm further the release mechanism of drug, the data was subjected to Korsmeyer’s-Peppas equation. The release exponent ‘n’ value was determined, based on ‘n’ value it can be explained that incorporated drug release by the anomalous (Non-Fickian) type of diffusion, involving swelling of the polymer matrix, as is evident by the slope values of more than 0.5 but less than 1 for the plot of log cumulative amount release Vs log time (Korsmeyer’s-Peppas plot). Except in case of formulation C5 which showed Non-Fickian super case II (slope = 1.180). When the average rate constant of the formulation were studied, it was observed that formulation C8 showed comparatively lower rates of release of drug, the results are shown in (Table 5). Among all the prepared films, C8 (chitosan/HPMC blend in the ratio of 75:25; cross-linked with sodium citrate) would be a better formulation based on the *in vitro* permeation studies as it released the drug in sustained release pattern for 24 hours without significantly releasing the drug in a burst manner in the initial hours. Among the non cross-linked formulation D3 would be a better formulation based on the *in vitro* permeation studies and first order rate constant.

**TABLE 5:** DRUG RELEASE KINETICS PARAMETERS OF RELEASE STUDIES THROUGH DIALYSIS MEMBRANE

Formulation Code	Cumulative% of drug release	First order Equation		Higuchi’s Equation	Korsmeyer’s-Peppas Equation
				
		R^2^	K	R^2^	n
D1	99.06±0.02	0.717	1.915×10^-1^	0.5742	0.8432
D2	101.95±0.44	0.9961	1.952×10^-1^	0.8486	0.655
D3	45.55±1.03	0.9184	2.326×10^-2^	0.9554	0.6292
D4	75.96±0.31	0.6757	5.527×10^-2^	0.7666	0.7158
D5	61.33±1.85	0.9575	3.661×10^-2^	0.9684	0.6226
D6	105.88±0.47	0.9846	2.643×10^-1^	0.8422	0.6403
D7	89.21±0.78	0.9258	8.797×10^-2^	0.8679	0.5776
D8	98.98±3.01	0.9987	1.966×10^-1^	0.8578	0.7462
C1	96.99±4.64	0.9767	1.473×10^-1^	0.8223	0.8995
C2	74.34±1.85	0.7839	5.734×10^-2^	0.8238	0.8293
C3	40.79±3.01	0.8825	1.980×10^-2^	0.9434	0.6747
C4	97.60±0.78	0.9659	1.648×10^-1^	0.9692	0.8641
C5	41.18±0.47	0.8758	2.233×10^-2^	0.9322	1.1804
C6	62.72±1.85	0.9742	4.306×10^-2^	0.9121	0.9764
C7	85.14±0.31	0.9939	8.175×10^-2^	0.9765	0.7609
C8	34.20±0.35	0.9925	1.658×10^-2^	0.9303	0.6454

**Fig. 5:**
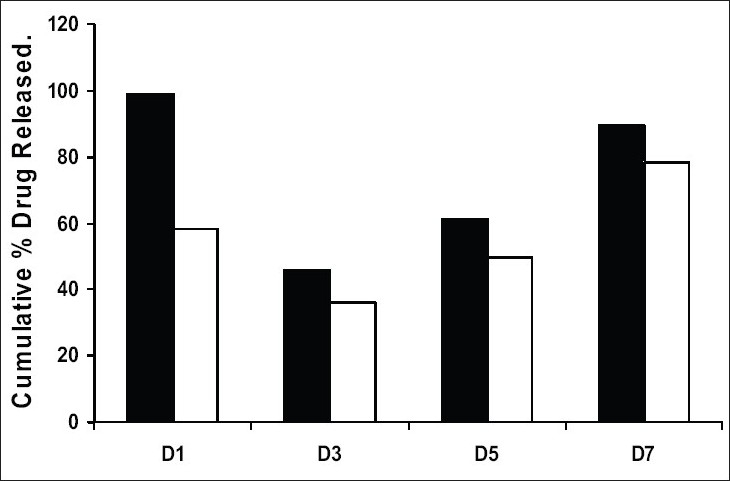
Drug release through dialysis membrane and rat skin. Comparison of drug release through dialysis membrane (■) and rat skin (□)

The skin permeation studies were carried out using rat skin. The apparatus for the study was arranged in the same manner as for dialysis membrane permeation study. The results are shown in the Table 6. As done in previous experiment, the drug permeation data was plotted (fig 5) according to first order, Higuchi’s and Korsemeyer-Peppa equation to know the release mechanisms. The formulations showed the fair linearity with respect to first order (R^2^ = 0.9843−0.9138) and Higuchi’s equations (R^2^ = 0.9696−0.9356) hence to confirm precisely the domination mechanism; the data was plotted according to Korsemeyer’s equation. The lines obtained were linear (R^2^ = 0.9461−0.9655), slope values vary between (0.6160 and 0.6792).

**TABLE 6:** DRUG RELEASE KINETICS PARAMETERS OF RELEASE STUDIES THROUGH RAT SKIN

Formulation Code	First order Equation		Higuchi’s Equation	Korsmeyer’s-Peppas Equation	
			
	R^2^	K		R^2^	n
D1	0.9843	3.5×10^-2^	0.9356	0.9655	0.6613
D3	0.9138	1.7×10^-2^	0.9687	0.9621	0.6792
D5	0.9453	2.6×10^-2^	0.9696	0.9461	0.6160
D7	0.9592	6.0×10^-2^	0.9494	0.9494	0.6670

In conclusion, it has been observed that diffusion is dominant mechanism for drug release following Non-Fickian type of diffusion. Among all the prepared films, D3 would be better formulation based on the *in vitro* skin permeation studies as it sustained the release of drug for longer duration with out significantly releasing the drug in a burst manner in the initial hours. Hence the present study demonstrates that Etoricoxib transdermal patches could be successfully prepared using chitosan, modified chitosan and chitosan-HPMC blend.
